# Overexpression of Dehydrogenase/Reductase 9 Predicts Poor Response to Concurrent Chemoradiotherapy and Poor Prognosis in Rectal Cancer Patients

**DOI:** 10.3389/pore.2022.1610537

**Published:** 2022-10-06

**Authors:** Tzu-Ju Chen, Bei-Hao Hsu, Sung-Wei Lee, Ching-Chieh Yang, Yu-Feng Tian, Yu-Hsuan Kuo, Wan-Shan Li, Hsin-Hwa Tsai, Li-Ching Wu, Cheng-Fa Yeh, Chia-Lin Chou, Hong-Yue Lai

**Affiliations:** ^1^ Department of Clinical Pathology, Chi Mei Medical Center, Tainan, Taiwan; ^2^ Department of Medical Technology, Chung Hwa University of Medical Technology, Tainan, Taiwan; ^3^ Department of General Surgery, Chung Shan Medical University Hospital, Taichung, Taiwan; ^4^ Department of Radiation Oncology, Chi Mei Medical Center, Liouying, Taiwan; ^5^ Department of Radiation Oncology, Chi Mei Medical Center, Tainan, Taiwan; ^6^ Department of Pharmacy, Chia-Nan University of Pharmacy and Science, Tainan, Taiwan; ^7^ Division of Colon and Rectal Surgery, Department of Surgery, Chi Mei Medical Center, Tainan, Taiwan; ^8^ Division of Hematology and Oncology, Department of Internal Medicine, Chi-Mei Medical Center, Tainan, Taiwan; ^9^ College of Pharmacy and Science, Chia Nan University, Tainan, Taiwan; ^10^ Department of Pathology, Chi Mei Medical Center, Tainan, Taiwan; ^11^ Institute of Biomedical Science, National Sun Yat-Sen University, Kaohsiung, Taiwan; ^12^ Department of Medical Research, Chi Mei Medical Center, Tainan, Taiwan; ^13^ Trans-Omic Laboratory for Precision Medicine, Precision Medicine Center, Chi Mei Medical Center, Tainan, Taiwan; ^14^ Department of Laboratory Medicine, China Medical University Hospital, Taichung, Taiwan; ^15^ Division of General Internal Medicine, Chi Mei Medical Center, Tainan, Taiwan; ^16^ Department of Environment Engineering and Science, Chia Nan University of Pharmacy and Science, Tainan, Taiwan; ^17^ Department of Pharmacology, School of Medicine, College of Medicine, China Medical University, Taichung, Taiwan

**Keywords:** biomarker, rectal cancer, *DHRS9*, epithelial cell differentiation, keratan sulfate, mucin

## Abstract

**Objective:** To reduce the risk of locoregional recurrence, the addition of neoadjuvant concurrent chemoradiotherapy (CCRT) is recommended before surgical management for rectal cancer patients. However, despite identical tumor histology, individual patient response to neoadjuvant CCRT varies greatly. Accordingly, a comprehensive molecular characterization that is used to predict CCRT efficacy is instantly needed.

**Methods:** Pearson’s chi-squared test was utilized to correlate dehydrogenase/reductase 9 (DHRS9) expression with clinicopathological features. Survival curves were created applying the Kaplan-Meier method, and the log-rank test was conducted to compare prognostic utility between high and low DHRS9 expression groups. Multivariate Cox proportional hazards regression analysis was applied to identify independent prognostic biomarkers based on variables with prognostic utility at the univariate level.

**Results:** Utilizing a public transcriptome dataset, we identified that the *DHRS9* gene is the most considerably upregulated gene related to epithelial cell differentiation (GO: 0030855) among rectal cancer patients with CCRT resistance. Employing immunohistochemical staining, we also demonstrated that high DHRS9 immunoexpression is considerably associated with an aggressive clinical course and CCRT resistance in our rectal cancer cohort. Among all variables with prognostic utility at the univariate level, only high DHRS9 immunoexpression was independently unfavorably prognostic of all three endpoints (all *p* ≤ 0.048) in the multivariate analysis. In addition, applying bioinformatic analysis, we also linked *DHRS9* with unrevealed functions, such as keratan sulfate and mucin synthesis which may be implicated in CCRT resistance.

**Conclusion:** Altogether, *DHRS9* expression may serve as a helpful predictive and prognostic biomarker and assist decision-making for rectal cancer patients who underwent neoadjuvant CCRT.

## Introduction

Stemming from the large intestine (colon) or rectum, colorectal cancer (CRC) ranks third in men and ranks second in women in terms of global cancer incidence (1). Notably, in Eastern Asia, rectal cancer incidence rates rank among the highest (1). Rectal cancer patients without distant metastasis are usually managed by a standardized surgical technique termed total mesorectal excision (2). To reduce the risk of locoregional recurrence, the use of multimodal approach such as neoadjuvant concurrent chemoradiotherapy (CCRT) is recommended before surgical management for rectal cancer patients with stage T3/T4 or node-positive (N1/N2) disease (2). However, despite identical tumor histology, individual patient response to neoadjuvant CCRT can range from complete remission to disease progression (3). Identification of predictive factors such as tumor regression (4) has been introduced for decision-making according to preoperative staging information, but a comprehensive molecular characterization that is used to predict individual therapy response remains lacking.

The intestinal epithelium is continuously regenerated (every 3–5 days) by a population of stem cells (5) that differentiate into multiple epithelial cell subsets which operate in concert to maintain the intestinal barrier and provide host defense. Among these epithelial cell subsets, goblet cells are well known for their secretion of the glycosylated mucin 2 (MUC2) protein, which creates the protective mucus barrier covering the epithelium (6). *Muc2*-deficient mice have been indicated to develop spontaneous colitis, an inflammatory bowel disease, caused by direct contact between the bacteria and the colonic epithelium (7). Low MUC2 expression was also correlated with poor survival in CRC patients without receiving radiation or chemotherapy (8, 9). However, our previous study has suggested that MUC2 overexpression is an unfavorable predictive and prognostic factor for rectal adenocarcinoma patients receiving neoadjuvant CCRT (10). These observations imply that the roles of mucin in tumor progression and CCRT efficacy may be different. In addition, poorly differentiated CRC with high proliferative and metastatic capacity is correlated with worse patient survival (11). Interestingly, it has been reported that, in the radio-resistant rectal cancer cells, most of the differently expressed genes were associated with cell-cell adhesion and regulation of epithelial cell differentiation (12). Whether epithelial cell differentiation can function as a barrier to defend against radiation penetration remains an open question. Accordingly, the role of epithelial cell differentiation in the efficacy of CCRT in rectal cancer patients and the underlying molecular mechanisms deserve further investigation.

Using a transcriptome dataset, we focused on differentially expressed genes in relation to epithelial cell differentiation and then identified that the dehydrogenase/reductase 9 (*DHRS9*) gene level was the most considerably upregulated among CCRT nonresponders in rectal cancer. The human *DHRS9* gene, which maps to chromosome 2q31.1, encodes an enzyme of the short-chain dehydrogenase/reductase (SDR) family. *DHRS9*, also known as retinol dehydrogenase 15 (RDH15), can catalyze the oxidation of retinol to retinaldehyde and is found specifically in the intestine and featured mainly by mucin production according to the analysis of single cell type expression cluster (https://www.proteinatlas.org/ENSG00000073737-DHRS9). DHRS9 is also implicated in the biosynthesis of all-trans-retinoic acid (atRA), which is the most active retinoid metabolite and contributes to the suppression of CRC progression through cell growth inhibition and cell differentiation induction (13). Moreover, low *DHRS9* expression has been indicated to be correlated with poor prognosis in CRC patients, but those who received any anti-cancer therapy were excluded (14). Consequently, the current study intended to connect *DHRS9* expression to CCRT efficacy and patient survival and illuminate the role of *DHRS9* in rectal cancer patients undergoing neoadjuvant CCRT.

## Patients and Methods

### Transcriptome Analysis of Rectal Cancer Biopsies

A public transcriptomic dataset (GSE35452) of tissue blocks from forty-six rectal adenocarcinoma patients receiving neoadjuvant CCRT was analyzed to recognize promising genes related to CCRT efficacy. Before receiving CCRT, rectal cancer biopsies were collected in the course of colonoscopic examination in this dataset. Applying the Affymetrix Human Genome U133 Plus 2.0 Array platform, the expression profiles were determined, and all probe sets were analyzed without any filtering. The tumor specimens were split into “nonresponders” and “responders” as determined by the response to CCRT, and a comparative analysis was accomplished under supervision. We took notice of differentially expressed genes in relation to epithelial cell differentiation (GO: 0030855) and then identified those with log2 ratio >0.2 and *p* less than 0.005 for further assessment.

### Patient Eligibility and Enrollment

Approved by the Ethics Committee and Institutional Review Board (IRB) of Chi Mei Medical Center (10302014), formalin-fixed paraffin-embedded (FFPE) tissue blocks of 172 consecutive (from 1998 to 2004) rectal cancer patients were obtained from our biobank. We retrospectively reviewed the medical records of these patients and recorded their clinical and pathological characteristics and clinical outcomes. All patients were primarily clinically diagnosed as having rectal cancer by colonoscopy and no distant metastasis by abdominopelvic computed tomography (CT) and/or chest X-ray radiography. The patients were regularly followed up following diagnosis until their last appointment or death. Before surgery, all patients were treated with CCRT, comprising 24-h continuous infusion of 5-fluorouracil (5-FU)-based chemotherapy and radiotherapy with a total dose of 45–50 Gy in twenty-five fractions over 5 weeks. Before or after CCRT, for patients with nodal or tumoral status no less than N1 or T3, respectively, adjuvant chemotherapy was performed. Almost all patients developed events within 60 months. For the non-eventful patients, the mean follow-up duration was 66.4 months (median 59.2, ranged from 10.3 to 131.3).

### Histopathological Assessment and Immunohistochemical Scoring

Blind to the clinical information of the patients, two expert pathologists (Wan-Shan Li and Tzu-Ju Chen) looked over all tumor specimens to gain more objective assessment. As stated by the eighth edition of the American Joint Committee on Cancer (AJCC) tumor-node-metastasis (TNM) staging system, the tumor and node stages were determined. The Dworak tumor regression grade system was used to predict the tumor response to CCRT and is defined as follows: 0–1, no or little response; 2–3, modest response; 4, complete response (4). Following standard protocols (15), immunohistochemical (IHC) staining was conducted. The slides were placed in an oven with 65°C to melt the paraffin and were deparaffinized in xylene and rehydrated. Heat-induced antigen retrieval was performed in 10 mM sodium citrate buffer (pH 6) in a microwave for 20 min. Subsequently, the slides were incubated with *DHRS9* primary antibody (H00010170-M05, 1:100) (Novus Biologicals, Littleton, CO, United States) for 1 h at room temperature and then stained with secondary antibody (Dako REAL™ EnVision™ Detection System, Peroxidase/DAB, Rabbit/Mouse) for 30 min at room temperature. The H-score provided a dynamic range to quantify the biomarker of interest from the IHC staining and was calculated based on the following equation: H-score = Σ*Pi* (*i* + 1), where *Pi* is the percentage, varying from 0% to 100%, of stained tumor cells for each intensity, and *i* is the intensity (0–3+) of staining. The H-score assigned an IHC score to each patient and comprised values from 100 to 400 (16). The median H-score was utilized to divide DHRS9 immunoreactivity into low and high expressions.

### Gene Function Prediction of the Cancer Genome Atlas Data

To forecast the unidentified functions of *DHRS9* in rectal cancer, the associations between the mRNA levels of *DHRS9* and its coexpressed genes from the colorectal adenocarcinoma dataset (*n* = 594, PanCancer Atlas, TCGA) were reviewed applying the cBioPortal online platform (http://cbioportal.org). Thereafter, the top two hundred transcripts with either a positive association or a negative association with *DHRS9* were annotated applying the Gene Ontology (GO) classification system (http://geneontology.org/) according to Protein Annotation Through Evolutionary Relationship (PANTHER) overrepresentation test and were ranked by fold enrichment. An R script with ggplot2 package was applied to visualize the representative GO terms.

### Statistical Analysis

The Statistical Product and Service Solutions (SPSS) software version 22.0 was employed for all statistical analyses. Pearson’s chi-squared test was utilized to correlate DHRS9 expression with clinicopathological features. Appraised from operation to the date when the event developed, three endpoints: metastasis-free survival (MeFS), local recurrence-free survival (LRFS), and disease-specific survival (DSS), were analyzed. Survival curves were created applying the Kaplan-Meier method, and the log-rank test was conducted to compare prognostic utility between high and low DHRS9 expression groups. Multivariate Cox proportional hazards regression analysis was applied to identify independent prognostic biomarkers based on variables with prognostic utility at the univariate level. A two-tailed test with *p* < 0.05 was regarded as statistical significance.

## Results

### *DHRS9* Upregulation is Linked to Concurrent Chemoradiotherapy Resistance in Rectal Adenocarcinoma Patients

**FIGURE 1:**
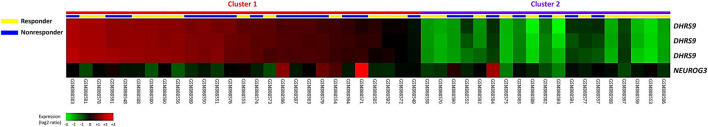
Transcriptome analysis of genes associated with epithelial cell differentiation and the response to CCRT. The expression levels of downregulated and upregulated genes are marked in green and red, respectively. We identified *DHRS9* as the most remarkably upregulated gene in relation to epithelial cell differentiation (GO: 0030855) among CCRT-resistant rectal cancer patients.

To recognize promising genes in relation to CCRT efficacy, a published transcriptomic dataset (GSE35452) of tissue blocks from forty-six rectal adenocarcinoma patients receiving neoadjuvant CCRT was analyzed. Twenty-two patients (47.8%) and 24 patients (52.2%) were allocated as nonresponders and responders, respectively, and a comparative analysis was carried out to identify predictive genetic biomarkers. To investigate the role and the underlying molecular mechanisms of epithelial cell differentiation in the efficacy of CCRT in patients with rectal cancer, we focused on epithelial cell differentiation (GO: 0030855) (Supplementary Table S1) and identified 4 probes covering 2 transcripts: *DHRS9* and neurogenin 3 (*NEUROG3*), associated with CCRT resistance (Table 1; Figure 1). Specifically found in the intestine and significantly upregulated among CCRT-resistant rectal cancer patients (log2 ratio = 1.3317, *p* = 0.0001), the *DHRS9* gene was selected for further analysis. As a consequence, we intended to appraise the utility of *DHRS9* expression status on CCRT efficacy, clinicopathological features, and patient prognosis in our rectal cancer cohort.

**TABLE 1:** Summary of differentially expressed genes associated with epithelial cell differentiation (GO: 0030855) in relation to CCRT resistance in rectal adenocarcinoma.

Probe	Comparison log ratio	Comparison *p*-Value	Gene symbol	Gene name	Biological process	Molecular function
207965_at	0.2712	0.0003	*NEUROG3*	Neurogenin 3	Cell differentiation, central nervous system development, epithelial cell differentiation, multicellular organismal development, nervous system development, peripheral nervous system development, positive regulation of transcription from RNA polymerase II promoter, regulation of transcription, regulation of transcription; DNA-dependent, transcription	DNA binding, transcription factor activity, transcription regulator activity
219799_s_at	0.9594	0.0035	*DHRS9*	Dehydrogenase/reductase (SDR family) member 9	9-cis-retinoic acid biosynthetic process, androgen metabolic process, epithelial cell differentiation, metabolic process, progesterone metabolic process, retinol metabolic process	3-alpha (17-beta)-hydroxysteroid dehydrogenase (NAD+) activity, alcohol dehydrogenase activity, oxidoreductase activity, racemase and epimerase activity, retinol dehydrogenase activity
223952_x_at	1.3317	0.0001	*DHRS9*	Dehydrogenase/reductase (SDR family) member 9	9-cis-retinoic acid biosynthetic process, androgen metabolic process, epithelial cell differentiation, metabolic process, progesterone metabolic process, retinol metabolic process	3-alpha (17-beta)-hydroxysteroid dehydrogenase (NAD+) activity, alcohol dehydrogenase activity, oxidoreductase activity, racemase and epimerase activity, retinol dehydrogenase activity
224009_x_at	1.2017	0.001	*DHRS9*	dehydrogenase/reductase (SDR family) member 9	9-cis-retinoic acid biosynthetic process, androgen metabolic process, epithelial cell differentiation, metabolic process, progesterone metabolic process, retinol metabolic process	3-alpha (17-beta)-hydroxysteroid dehydrogenase (NAD+) activity, alcohol dehydrogenase activity, oxidoreductase activity, racemase and epimerase activity, retinol dehydrogenase activity

### Clinicopathological Characteristics of a Cohort of Rectal Cancer Patients

Tissue specimens of 172 rectal cancer patients treated with neoadjuvant CCRT were obtained from our biobank, and their clinical and pathological features are exhibited in Table 2. In the course of primary clinical diagnosis, the tumor status of 81 patients (47.1%) was early stage (cT1–T2), and the lymph node status of 125 patients (72.7%) was negative (cN0). After CCRT, 123 patients (71.5%) had no lymph node involvement (ypN0), and 86 patients (50%) had an invasion depth limited to the muscularis propria (ypT1–T2). In addition, no vascular invasion and perineural invasion was observed in 157 patients (91.3%) and 167 patients (97.1%), correspondingly. Also, the tumor regression grade was used to assess the therapeutic response in patients with rectal cancer following CCRT, and the results revealed that 37 patients (21.5%) had little or no response (grade 0–1).

**TABLE 2:** Associations between DHRS9 expression and clinicopathological features in 172 rectal adenocarcinoma patients undergoing neoadjuvant CCRT.

Parameter		No. of case	DHRS9 expression		*p*-Value
			Low exp	High exp	
Gender	Male	108	56	52	0.636
	Female	64	30	34	
Age	<70	106	52	54	0.875
	≧70	66	34	32	
Pre-Tx tumor status (Pre-T)	T1–T2	81	48	33	0.032*
	T3–T4	91	38	53	
Pre-Tx nodal status (Pre-N)	N0	125	68	57	0.086
	N1–N2	47	18	29	
Post-Tx tumor status (Post-T)	T1–T2	86	56	30	<0.001*
	T3–T4	86	30	56	
Post-Tx nodal status (Post-N)	N0	123	68	55	0.042*
	N1–N2	49	18	31	
Vascular invasion	Absent	157	84	73	0.005*
	Present	15	2	13	
Perineural invasion	Absent	167	85	82	0.368
	Present	5	1	4	
Tumor regression grade	Grade 0–1	37	10	27	<0.001*
	Grade 2–3	118	61	57	
	Grade 4	17	15	2	

Tx, treatment; *, statistically significant (bold values).

### Immunoexpression of DHRS9 and its Connections With Clinicopathological Parameters

**FIGURE 2:**
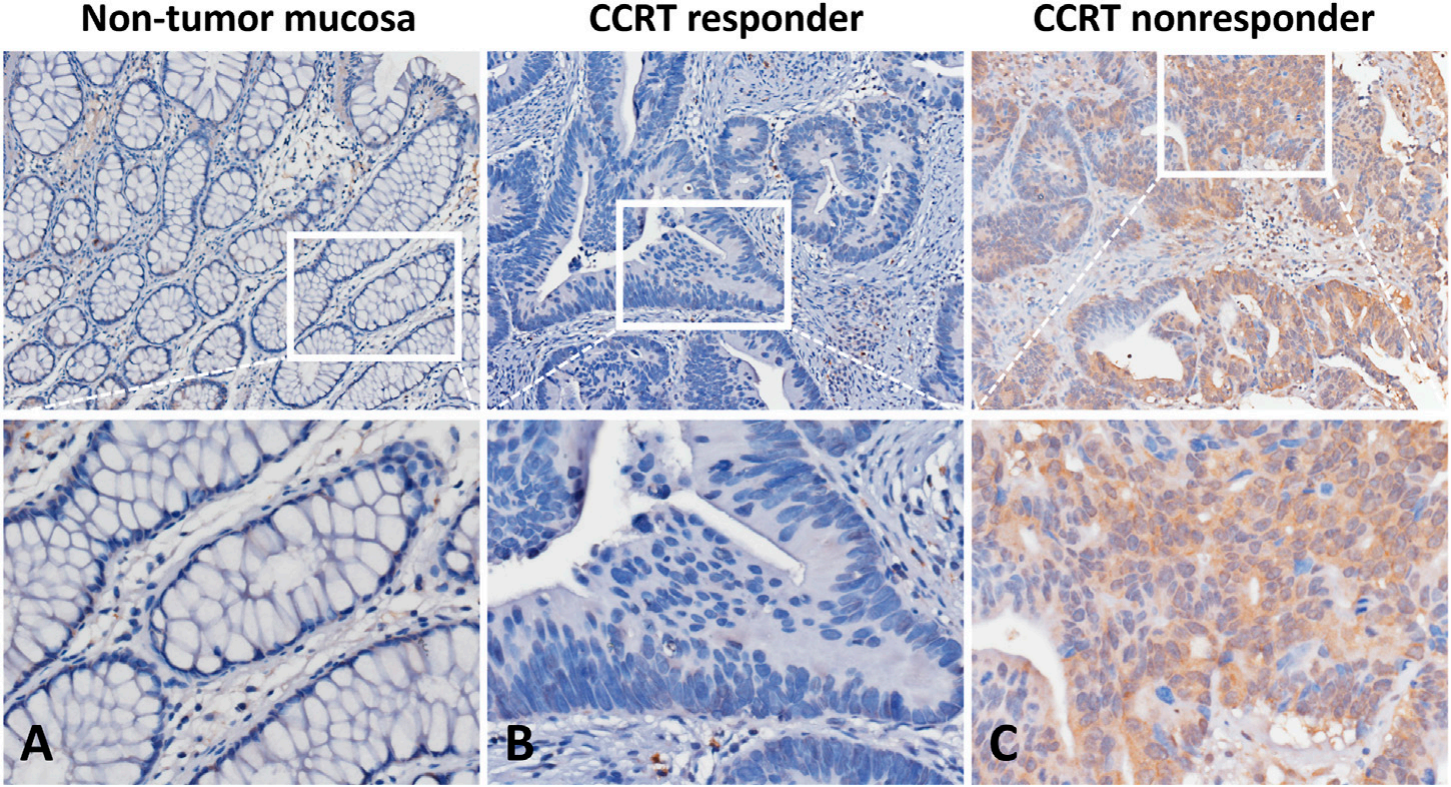
Immunohistochemical staining of DHRS9. **(A)** Non-tumor mucosa displayed no DHRS9 immunoexpression. Rectal cancer specimens presented **(B)** low DHRS9 immunoexpression in patients who responded to CCRT and **(C)** high DHRS9 immunoexpression in patients with CCRT resistance.

IHC staining was conducted to assess the utility of DHRS9 expression status on CCRT efficacy and clinicopathological parameters. As displayed in Table 2, high DHRS9 immunoexpression was remarkably connected to advanced pre-CCRT and post-CCRT tumor status (*p* = 0.032 and *p* < 0.001), post-CCRT lymph node involvement (*p* = 0.042), and vascular invasion (*p* = 0.005). Especially, tumors with high DHRS9 immunoexpression (H-scores were above or identical to the median of all scored cases) (Supplementary Figure S1) had a considerably lower grade of tumor regression (*p* < 0.001). Among patients with high DHRS9 immunoexpression, 27 patients (31.4%) had little or no response to CCRT (grade 0–1). Also, the representative images of IHC staining revealed that the immunoexpression of DHRS9 was remarkably higher in rectal cancer patients with CCRT resistance (Figures 2A–C).

### Prognostic Influence of DHRS9 Immunoexpression on Rectal Cancer Patients

**FIGURE 3:**
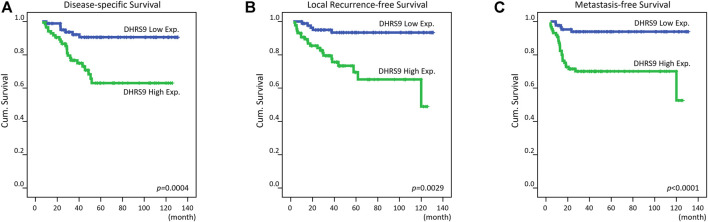
Kaplan–Meier survival analysis. Kaplan–Meier curves were created and showed that high DHRS9 immunoexpression was considerably correlated with worse **(A)** disease-specific survival, **(B)** local recurrence-free survival, and **(C)** metastasis-free survival.

Thirty-one patients (18%) died because of rectal adenocarcinoma, and local recurrence and distant metastasis were first detected in 27 patients (15.7%) and 31 patients (18%), respectively. Univariate and multivariate analyses were then carried out to assess the influence of clinicopathological features and DHRS9 immunoexpression on patient survival. The results of univariate analysis revealed that the advanced post-CCRT tumor status, low grade of tumor regression, and high DHRS9 immunoexpression (Figure 3) were considerably unfavorably prognostic of all three endpoints (all *p* ≤ 0.009): metastasis-free survival (MeFS), local recurrence-free survival (LRFS), and disease-specific survival (DSS) (Table 3). Furthermore, only high DHRS9 immunoexpression was independently unfavorably prognostic of all three endpoints (all *p* ≤ 0.048) in the multivariate analysis (Table 4).

**TABLE 3:** Univariate log-rank analysis for crucial clinicopathological factors and DHRS9 expression.

Parameter		No. of case	DSS		LRFS		MeFS	
			No. of event	*p*-Value	No. of event	*p*-Value	No. of event	*p*-Value
Gender	Male	108	20	0.9026	7	0.2250	17	0.3520
	Female	64	11		20		14	
Age	<70	106	19	0.8540	18	0.6615	20	0.7427
	≧70	66	12		9		11	
Pre-Tx tumor status (Pre-T)	T1–T2	81	10	0.0776	10	0.2261	11	0.1745
	T3–T4	91	21		17		20	
Pre-Tx nodal status (Pre-N)	N0	125	19	0.0711	15	0.0070*	19	0.0973
	N1–N2	47	21		12		12	
Post-Tx tumor status (Post-T)	T1–T2	86	7	0.0006*	7	0.0040*	8	0.0033*
	T3–T4	86	24		20		23	
Post-Tx nodal status (Post-N)	N0	123	21	0.5998	16	0.1320	20	0.4634
	N1–N2	49	10		11		11	
Vascular invasion	Absent	157	25	0.0184*	21	0.0028*	27	0.4470
	Present	15	6		6		4	
Perineural invasion	Absent	167	29	0.2559	25	0.0940	30	0.9083
	Present	5	2		2		1	
Tumor regression grade	Grade 0–1	37	13	0.0038*	10	0.0090*	14	0.0006*
	Grade 2–3	118	17		17		16	
	Grade 4	17	1		0		1	
DHRS9 expression	Low Exp	86	7	<0.0001*	5	0.0029*	5	<0.0001*
	High Exp	86	24		22		26	

DSS, disease-specific survival; LRFS, local recurrence-free survival; MeFS, metastasis-free survival; *, statistically significant (bold values).

**TABLE 4:** Multivariate analysis.

Parameter	DSS			LRFS			MeFS		
	H.R.	95% CI	*p*-Value	H.R.	95% CI	*p*-Value	H.R.	95% CI	*p*-Value
Tumor regression grade	1.901	0.936–3.861	0.076	2.058	0.957–4.425	0.065	**2.262**	**1.135**–**4.505**	0.020*
DHRS9 expression	**2.480**	**1.007**–**6.104**	0.048*	**3.423**	**1.216**–**9.637**	0.020*	**4.616**	**1.697**–**12.560**	0.003*
Vascular invasion	1.874	0.719–4.881	0.199	1.687	0.605–4.703	0.318	—	—	—
Post-Tx tumor status (Post-T)	**2.442**	**1.015**–**5.878**	0.046*	1.981	0.852–6.605	0.163	0.991	0.341–2.879	0.986
Pre-Tx nodal status (Pre-N)	—	—	—	1.889	0.773–4.618	0.112	—	—	—

DSS, disease-specific survival; LRFS, local recurrence-free survival; MeFS, metastasis-free survival; *, statistically significant (bold values).

### Function Prediction of *DHRS9 via* Bioinformatic Analysis

**FIGURE 4:**
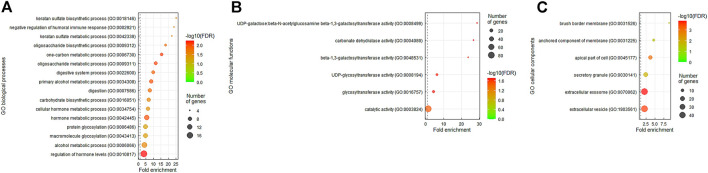
The Gene Ontology (GO) terms enriched in *DHRS9* overexpression. The genes that were coexpressed with *DHRS9* in the colorectal adenocarcinoma dataset (*n* = 594) from The Cancer Genome Atlas (TCGA) database were obtained employing the cBioPortal online platform (http://cbioportal.org). The top 200 genes co-upregulated with *DHRS9* were further analyzed applying the GO classification system (http://pantherdb.org) based on **(A)** biological processes, **(B)** molecular functions, or **(C)** cellular components and ranked by *fold enrichment* for functional annotation.

Since *DHRS9* has been considered as a moonlighting protein, we carried out a *gene* coexpression analysis to forecast the unidentified functions of *DHRS9* in rectal cancer. The top two hundred differentially expressed genes showing positive correlations (Supplementary Table S2) or negative correlations (Supplementary Table S3) with *DHRS9* were obtained from the colorectal adenocarcinoma dataset (*n* = 594, PanCancer Atlas, TCGA). Thereafter, the Gene Ontology (GO) classification system was employed for functional annotation on the basis of Protein Annotation Through Evolutionary Relationship (PANTHER) overrepresentation test, which compares a test gene list with a reference gene list and determines whether a particular group (e.g., biological process, molecular function, and cellular component) of genes is overrepresented. The results revealed that the most outstanding terms positively correlated with *DHRS9* were keratan sulfate biosynthetic process (GO: 0018146, fold enrichment: 25.24) and UDP-galactose:beta-*N*-acetylglucosamine beta-1,3-galactosyltransferase activity (GO: 0008499, fold enrichment: 28.84) in the matter of biological processes and molecular functions, correspondingly (Figures 4A,B). Especially, we identified that both the UDP-GlcNAc:betaGal beta-1,3-*N*-acetylglucosaminyltransferase 6 (*B3GNT6*) gene (Spearman’s correlation: 0.497) and the *B3GNT7* gene (Spearman’s correlation: 0.463) were implicated in both the functions mentioned above (Supplementary Figures S2A–B). The carbohydrate sulfotransferase 5 (*CHST5*) gene (Spearman’s correlation: 0.373) was involved only in the keratan sulfate biosynthetic process (Supplementary Figure S2C). Besides, as to cellular components (Figure 4C), the most prominent term positively correlated with *DHRS9* was brush border membrane (GO: 0031526, fold enrichment: 9.01). Interestingly, *MUC2* (Spearman’s correlation: 0.462) was also identified as one of the significant genes positively correlated with *DHRS9* (Supplementary Figure S2D).

## Discussion

All-trans-retinoic acid (atRA), an active metabolite of vitamin A (retinol), regulates gene expression through binding to the nuclear receptors and is applied as a classical differentiation therapy for acute promyelocytic leukemia (APL) (17). Since atRA has been reported to exert tumor-suppressive functions in various cancer types (18), *DHRS9*, implicated in the biosynthesis of atRA, may be considered to have antitumor activities. Actually, *DHRS9* downregulation has been correlated with poor survival in oral squamous cell carcinoma (19) and colorectal cancer (14) patients, but those who received any anti-cancer therapy were excluded in these two studies. Moreover, in pancreatic cancer, *DHRS9* overexpression has been correlated with poor prognosis (20), implying that *DHRS9* may play an oncogenic role based on distinct contexts. Interestingly, in the present study, we identified *DHRS9* as the most significantly upregulated gene in relation to epithelial cell differentiation among CCRT-resistant rectal cancer patients. In our rectal cancer cohort, we also demonstrated that high DHRS9 immunoexpression is remarkably linked to poor therapeutic response to CCRT and inferior patient survival, suggesting that epithelial cell differentiation may play a role in reducing CCRT efficacy in patients with rectal cancer.

To identify the unrevealed functions of *DHRS9* in rectal cancer, we carried out a *gene* coexpression analysis and found that the most significant GO terms positively correlated with *DHRS9* were keratan sulfate biosynthetic process and UDP-galactose:beta-*N*-acetylglucosamine beta-1,3-galactosyltransferase activity (Figures 4A,B). Glycosaminoglycans (GAGs) are linear polysaccharides with highly negative charges and display distinct functions as determined by their molecular weight and sulfation degree. In spite of their complicated structure, the backbone of these glycans is generally simply composed of repeating disaccharide units containing alternating hexosamines (glucosamine or galactosamine) and uronic acids (glucuronic or iduronic acid) (21). Based on the combination of different amino sugars and uronic acids, GAGs are categorized into four primary groups, including keratan sulfate (KS), which alternates between *N*-acetylglucosamine (GlcNAc) and galactose (Gal) and does not contain uronic acids (22). GAGs, the major macromolecules in the extracellular matrix (ECM), may function as a protective barrier that impedes drug delivery to the tumor cells (23). Additionally, GAGs can also play a key role in cell signaling and modulate abundant biological functions (24). Recent evidence suggests that GAGs may be involved in atRA-induced neural differentiation (25) and cancer stem cell formation and therapeutic resistance (26). Keratan sulfate is also regarded as a glycan marker expressed by stem cells (27), which is suggested to confer drug resistance in CRC patients (28). Nevertheless, the correlations among *DHRS9* expression, GAGs, especially keratan sulfate, and CCRT resistance in rectal cancer patients need to be further investigated.

Proteoglycans (PGs) are made up of a core protein and at least one covalently attached GAG side chain. Keratan sulfate is the newest GAG based on an evolutionary perspective but the least understood. On the basis of linkage structure utilized to attach to PG core proteins, internal structural organization, and tissue distribution, keratan sulfate can be classified into three types, namely KS type I (corneal KS, *N*-linked), II (skeletal KS, *O*-linked), and III (brain KS, *O*-linked) (29). Especially, KS type II attaches to a threonine or serine (Thr/Ser) residue on the core protein *via* an *O*-linked mucin-type structure (core 2, GalNAc-Thr/Ser). During the biosynthesis of KS, several glycosyltransferases and sulfotransferases act to add Gal or GlcNAc to an acceptor residue for chain elongation and undergo sulfation at carbon position 6 (C6) on Gal-GlcNAc either individually or collectively, respectively (30). The chain length and sulfation degree (charge heterogeneity) of KS increase with the age of the connective tissues where it is enriched in and their pathological status, including tumor development. Intriguingly, we observed that the *B3GNT6* and *B3GNT7* genes (Supplementary Figures S2A–B), which encode N-acetylglucosaminyltransferase enzymes, and the carbohydrate sulfotransferase 5 (CHST5) gene (Supplementary Figure S2C) were positively correlated with *DHRS9*.

B3GNT6 supports the formation of an *O*-linked mucin-type core 3 structure (31), and B3GNT7 is responsible for elongation of KS chains (32). In addition, CHST5 is a sulfotransferase that can transfer sulfate to *O*-linked glycans of mucin-type glycoproteins (33). Actually, the GalNAc residue of mucin-type glycoproteins can also serve as an acceptor molecule for the addition of Gal or GlcNAc that can also be sulfated and contribute to minimally sulfated KS type II-like mucins (34). As a key component of the ECM, mucins create a gel-like epithelial barrier thought to protect the gut lumen from external stress and microbial infection. However, aberrant mucin synthesis may also function as a barrier to drug penetration and cytotoxic T cell infiltration (35). MUC2, the major intestinal mucin, has been suggested to carry immunoregulatory signals to favor tumor growth in the large intestine (36), and our previous study also demonstrated that overexpression of MUC2 is linked to CCRT resistance and poor prognosis in patients with rectal adenocarcinoma (10). Notably, we also identified that the *MUC2* gene was positively correlated with *DHRS9* in *gene* coexpression analysis (Supplementary Figure S2D). In addition, brush border membrane was the most prominent term positively correlated with *DHRS9* in terms of cellular components (Figure 4C). The epithelium of the villus is composed primarily of absorptive enterocytes and mucin-secreting goblet cells. Since the brush border is formed with microvilli on the apical surface of the enterocytes, whether *DHRS9* can promote mucin synthesis through enterocyte–goblet cell interaction to defend against CCRT penetration deserves further analysis.

To predict the tumor response to preoperative CCRT, the tumor regression grade system is commonly used based on pathological features observed in surgical specimens. However, before surgery, there is currently no precise tool to predict CCRT effectiveness. Benefitting from the advancement of sequencing technologies, only small piece of tissue is required to obtain genetic information. To exploit our findings in the clinical practice, rectal cancer biopsies could be collected in the course of colonoscopic examination before receiving neoadjuvant CCRT. Afterwards, these biopsies could be used to conduct RNA sequencing or apply array platform and guide treatment more accurately according to the biomarkers such as high *DHRS9* level detected.

## Conclusion

The intestinal epithelium is composed of multiple well-differentiated epithelial cell subsets which work in concert to maintain the intestinal barrier and provide host defense. However, aberrant epithelial cell differentiation may be associated with therapy resistance. In this study, we identified that *DHRS9* is the most significantly upregulated gene related to epithelial cell differentiation among CCRT-resistant rectal cancer patients. We also demonstrated that high DHRS9 immunoexpression is considerably associated with an advanced disease stage, CCRT resistance, and inferior prognosis in patients with rectal adenocarcinoma. Additionally, we also linked *DHRS9* with unrevealed functions, such as keratan sulfate and mucin synthesis which may be implicated in CCRT resistance. Collectively, *DHRS9* expression may assist decision-making for rectal cancer patients who underwent neoadjuvant CCRT.

## Data Availability

The datasets presented in this study can be found in online repositories. The names of the repository/repositories and accession number(s) can be found in the article/Supplementary Material.
