# Supplementary figures and images for: Overexpression of Dehydrogenase/Reductase 9 Predicts Poor Response to Concurrent Chemoradiotherapy and Poor Prognosis in Rectal Cancer Patients
Source: Pathol Oncol Res. 2022 Oct 6;28:1610537. doi: 10.3389/pore.2022.1610537 (PMC9582124; doi:10.3389/pore.2022.1610537)

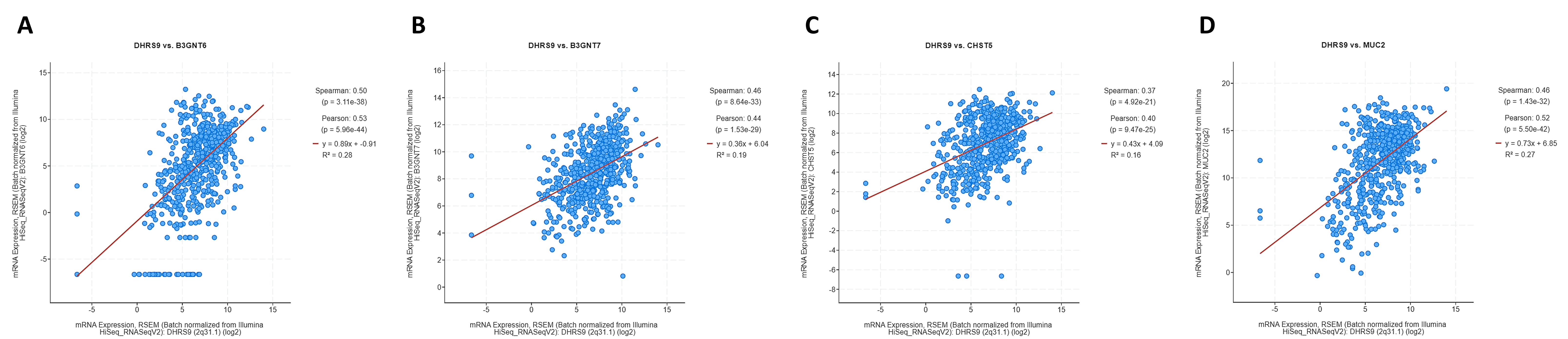

Supplement: Supplementary file 1 [file Image2.TIF]

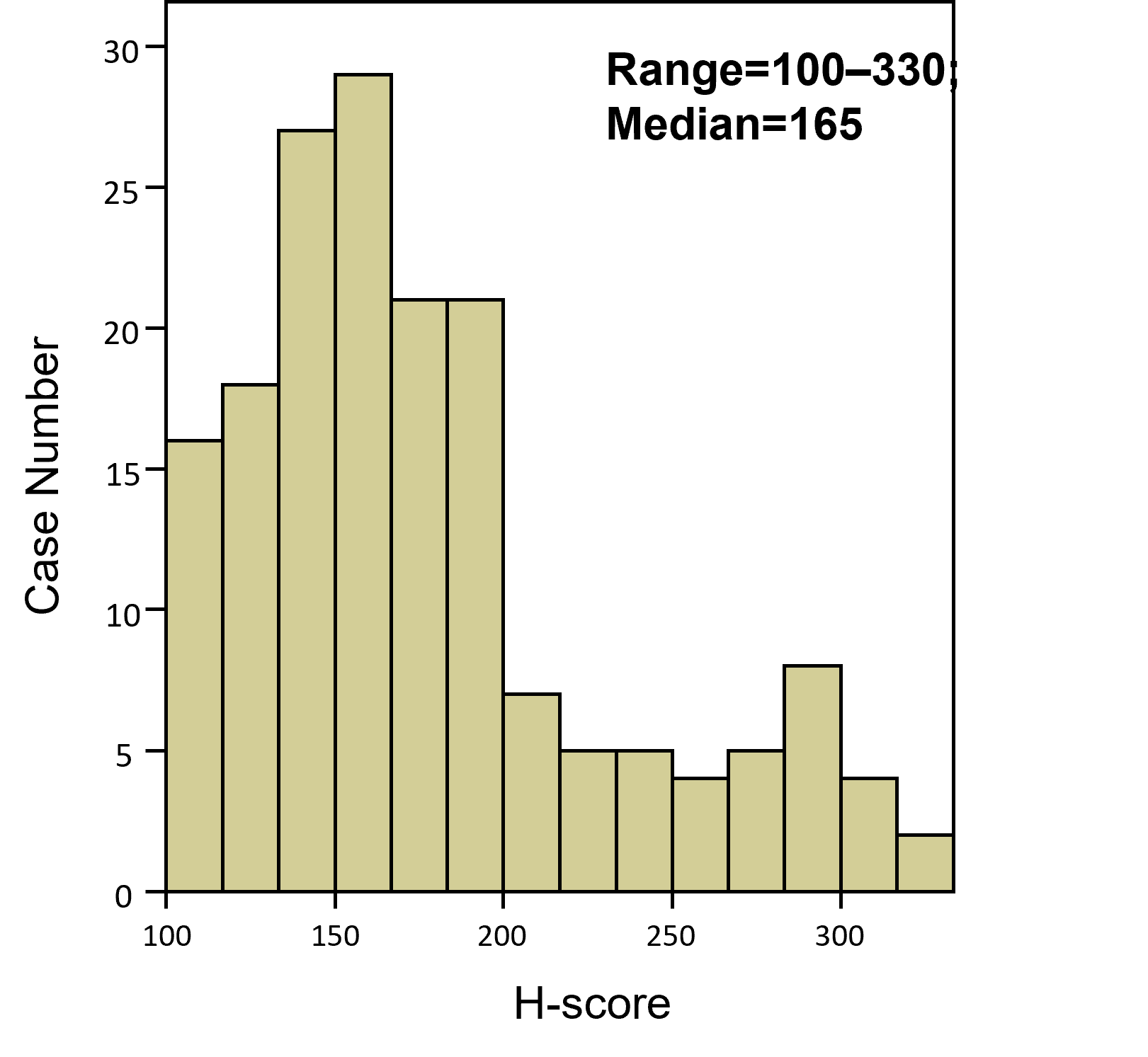

Supplement: Supplementary file 2 [file Image1.TIF]
