# Supplementary material for: Overexpression of Dehydrogenase/Reductase 9 Predicts Poor Response to Concurrent Chemoradiotherapy and Poor Prognosis in Rectal Cancer Patients
Source: Pathol Oncol Res. 2022 Oct 6;28:1610537. doi: 10.3389/pore.2022.1610537 (PMC9582124; doi:10.3389/pore.2022.1610537)
Supplement: Supplementary file 3 [file DataSheet1.docx]

**Supplementary Table 1. A list of the genes related to epithelial cell differentiation (GO: 0030855).**

| **Probe** | **Gene Symbol** | **Gene Name** | **Comparison Log Ratio (nonresponder vs. responder)** | **Comparison *p*-Value** |
| --- | --- | --- | --- | --- |
| 207965_at | NEUROG3 | neurogenin 3 | 0.2712 | 0.0003 |
| 219799_s_at | DHRS9 | dehydrogenase/reductase (SDR family) member 9 | 0.9594 | 0.0035 |
| 223952_x_at | DHRS9 | dehydrogenase/reductase (SDR family) member 9 | 1.3317 | 0.0001 |
| 224009_x_at | DHRS9 | dehydrogenase/reductase (SDR family) member 9 | 1.2017 | 0.001 |
| 201510_at | ELF3 | E74-like factor 3 (ets domain transcription factor; epithelial-specific ) | 0.2004 | 0.1242 |
| 204451_at | FZD1 | frizzled homolog 1 (Drosophila) | 0.0634 | 0.8818 |
| 204452_s_at | FZD1 | frizzled homolog 1 (Drosophila) | -0.1193 | 0.1752 |
| 204667_at | FOXA1 | forkhead box A1 | 0.0999 | 0.7647 |
| 205185_at | SPINK5 | serine peptidase inhibitor; Kazal type 5 | 0.8647 | 0.0349 |
| 205817_at | SIX1 | SIX homeobox 1 | -0.0334 | 0.5091 |
| 206300_s_at | PTHLH | parathyroid hormone-like hormone | -0.0512 | 0.578 |
| 206771_at | UPK3A | uroplakin 3A | 0.0773 | 0.3862 |
| 207826_s_at | ID3 | inhibitor of DNA binding 3; dominant negative helix-loop-helix protein | 0.2989 | 0.0505 |
| 207862_at | UPK2 | uroplakin 2 | -0.0076 | 0.8839 |
| 208250_s_at | DMBT1 | deleted in malignant brain tumors 1 | -0.0241 | 0.9441 |
| 208510_s_at | PPARG | peroxisome proliferator-activated receptor gamma | 0.2079 | 0.249 |
| 210064_s_at | UPK1B | uroplakin 1B | -0.0865 | 0.4921 |
| 210065_s_at | UPK1B | uroplakin 1B | -0.1297 | 0.2763 |
| 210103_s_at | FOXA2 | forkhead box A2 | 0.326 | 0.1966 |
| 210220_at | FZD2 | frizzled homolog 2 (Drosophila) | -0.0221 | 0.9252 |
| 210355_at | PTHLH | parathyroid hormone-like hormone | -0.0295 | 0.8927 |
| 210827_s_at | ELF3 | E74-like factor 3 (ets domain transcription factor; epithelial-specific ) | 0.1195 | 0.3699 |
| 211756_at | PTHLH | parathyroid hormone-like hormone | -0.1386 | 0.3359 |
| 213240_s_at | KRT4 | keratin 4 | 0.1337 | 0.5262 |
| 214312_at | FOXA2 | forkhead box A2 | 0.0026 | 0.9692 |
| 214399_s_at | KRT4 | Keratin 4 | -0.0161 | 0.7428 |
| 214624_at | UPK1A | uroplakin 1A | 0.0417 | 0.5087 |
| 217325_at | KRT3 | keratin 3 | -0.0025 | 0.9636 |
| 219845_at | BARX1 | BARX homeobox 1 | 0.1024 | 0.2817 |
| 219850_s_at | EHF | ets homologous factor | 0.065 | 0.7547 |
| 222932_at | EHF | ets homologous factor | -0.0096 | 0.9581 |
| 224189_x_at | EHF | ets homologous factor | 0.1374 | 0.6001 |
| 225645_at | EHF | Ets homologous factor | 0.0805 | 0.7192 |
| 228347_at | SIX1 | SIX homeobox 1 | -0.0461 | 0.5884 |
| 230911_at | SIX1 | SIX homeobox 1 | 0.0325 | 0.5452 |
| 232360_at | EHF | ets homologous factor | 0.3339 | 0.1126 |
| 232361_s_at | EHF | ets homologous factor | 0.0745 | 0.7478 |
| 237086_at | FOXA1 | Forkhead box A1 | 0.2144 | 0.1195 |
| 40284_at | FOXA2 | forkhead box A2 | 0.2792 | 0.2287 |

**Supplementary Table 2. The top 200 genes positively correlated with DHRS9.**

| **Correlated Gene** | **Cytoband** | **Spearman's Correlation** | **p-Value** | **q-Value** |
| --- | --- | --- | --- | --- |
| **CLCA4** | 1p22.3 | 0.769 | 1.53E-116 | **3.05E-112** |
| **C11ORF86** | 11q13.2 | 0.74 | 6.94E-104 | **6.89E-100** |
| **CA2** | 8q21.2 | 0.686 | 2.29E-83 | **1.51E-79** |
| **CEACAM7** | 19q13.2 | 0.666 | 3.53E-77 | **1.76E-73** |
| **TSPAN1** | 1p34.1 | 0.642 | 5.02E-70 | **1.99E-66** |
| **ZG16** | 16p11.2 | 0.631 | 3.90E-67 | **1.29E-63** |
| **AKR1B10** | 7q33 | 0.627 | 5.22E-66 | **1.48E-62** |
| **ADTRP** | 6p24.1 | 0.611 | 7.16E-62 | **1.78E-58** |
| **PLAC8** | 4q21.22 | 0.609 | 2.32E-61 | **5.13E-58** |
| **B3GALT5** | 21q22.2 | 0.609 | 2.61E-61 | **5.18E-58** |
| **CA4** | 17q23.1 | 0.606 | 9.93E-61 | **1.79E-57** |
| **GCNT3** | 15q22.2 | 0.584 | 1.75E-55 | **2.90E-52** |
| **ABCC13** | 21q11.2 | 0.583 | 3.28E-55 | **5.01E-52** |
| **DUOX2** | 15q21.1 | 0.582 | 6.88E-55 | **9.77E-52** |
| **XDH** | 2p23.1 | 0.581 | 8.60E-55 | **1.14E-51** |
| **AHCYL2** | 7q32.1 | 0.58 | 1.94E-54 | **2.41E-51** |
| **TRIM40** | 6p22.1 | 0.57 | 2.13E-52 | **2.49E-49** |
| **CDKN2B-AS1** | 9p21.3 | 0.569 | 4.52E-52 | **4.98E-49** |
| **DUOXA2** | 15q21.1 | 0.561 | 2.31E-50 | **2.41E-47** |
| **MALL** | 2q13 | 0.557 | 1.44E-49 | **1.43E-46** |
| **MMP28** | 17q12 | 0.556 | 2.96E-49 | **2.80E-46** |
| **SDCBP2** | 20p13 | 0.55 | 3.37E-48 | **3.04E-45** |
| **ITLN1** | 1q23.3 | 0.548 | 1.30E-47 | **1.12E-44** |
| **HSD17B2** | 16q23.3 | 0.546 | 2.58E-47 | **2.14E-44** |
| **CA7** | 16q22.1 | 0.544 | 8.16E-47 | **6.48E-44** |
| **JCHAIN** | 4q13.3 | 0.542 | 1.93E-46 | **1.47E-43** |
| **B3GALT1** | 2q24.3 | 0.54 | 4.23E-46 | **3.12E-43** |
| **C2ORF88** | 2q32.2 | 0.54 | 4.52E-46 | **3.21E-43** |
| **ABCB11** | 2q31.1 | 0.653 | 8.20E-46 | **5.61E-43** |
| **VSIG2** | 11q24.2 | 0.532 | 1.65E-44 | **1.09E-41** |
| **CCL28** | 5p12 | 0.531 | 1.92E-44 | **1.23E-41** |
| **CD177** | 19q13.31 | 0.531 | 2.80E-44 | **1.74E-41** |
| **GPR15** | 3q11.2 | 0.523 | 7.92E-43 | **4.77E-40** |
| **PYY** | 17q21.31 | 0.515 | 2.05E-41 | **1.20E-38** |
| **GPT** | 8q24.3 | 0.507 | 4.52E-40 | **2.57E-37** |
| **SLC4A4** | 4q13.3 | 0.507 | 5.16E-40 | **2.85E-37** |
| **GUCA2B** | 1p34.2 | 0.616 | 1.41E-39 | **7.59E-37** |
| **CTSE** | 1q32.1 | 0.504 | 1.58E-39 | **8.24E-37** |
| **KRT20** | 17q21.2 | 0.504 | 1.74E-39 | **8.88E-37** |
| **HHLA2** | 3q13.13 | 0.504 | 1.90E-39 | **9.43E-37** |
| **MYPN** | 10q21.3 | 0.501 | 6.58E-39 | **3.19E-36** |
| **BRINP3** | 1q31.1 | 0.501 | 7.06E-39 | **3.34E-36** |
| **PKIB** | 6q22.31 | 0.499 | 1.30E-38 | **5.97E-36** |
| **FCGBP** | 19q13.2 | 0.499 | 1.32E-38 | **5.97E-36** |
| **B3GNT6** | 11q13.5 | 0.497 | 3.11E-38 | **1.37E-35** |
| **CASP5** | 11q22.3 | 0.497 | 3.34E-38 | **1.44E-35** |
| **FAM177B** | 1q41 | 0.494 | 8.54E-38 | **3.61E-35** |
| **GDPD2** | Xq13.1 | 0.493 | 1.45E-37 | **5.98E-35** |
| **FER1L6** | 8q24.13 | 0.492 | 2.21E-37 | **8.97E-35** |
| **BEST2** | 19p13.13 | 0.49 | 4.39E-37 | **1.75E-34** |
| **C15ORF48** | 15q21.1 | 0.489 | 6.41E-37 | **2.50E-34** |
| **VSIG1** | Xq22.3 | 0.488 | 8.04E-37 | **3.07E-34** |
| **GPAT3** | 4q21.23 | 0.488 | 8.70E-37 | **3.26E-34** |
| **LIMA1** | 12q13.12 | 0.486 | 2.27E-36 | **8.35E-34** |
| **REG4** | 1p12 | 0.485 | 2.79E-36 | **1.01E-33** |
| **ADGRF1** | 6p12.3\|6 | 0.483 | 6.29E-36 | **2.23E-33** |
| **KLF4** | 9q31.2 | 0.482 | 7.96E-36 | **2.74E-33** |
| **CIDEC** | 3p25.3 | 0.482 | 7.99E-36 | **2.74E-33** |
| **CHGA** | 14q32.12 | 0.482 | 8.22E-36 | **2.77E-33** |
| **MS4A12** | 11q12.2 | 0.591 | 8.73E-36 | **2.89E-33** |
| **SLC51B** | 15q22.31 | 0.481 | 1.14E-35 | **3.73E-33** |
| **MXD1** | 2p13.3 | 0.48 | 1.66E-35 | **5.33E-33** |
| **CLCA3P** | 1p22.3 | 0.479 | 2.72E-35 | **8.59E-33** |
| **AKR1B15** | 7q33 | 0.478 | 4.55E-35 | **1.41E-32** |
| **LGALS3** | 14q22.3 | 0.477 | 4.98E-35 | **1.52E-32** |
| **GDPD3** | 16p11.2 | 0.475 | 1.26E-34 | **3.81E-32** |
| **B4GALNT2** | 17q21.32 | 0.475 | 1.38E-34 | **4.08E-32** |
| **ARL14** | 3q25.33 | 0.475 | 1.42E-34 | **4.14E-32** |
| **RHOF** | 12q24.31 | 0.472 | 3.15E-34 | **9.06E-32** |
| **PTPRH** | 19q13.42 | 0.472 | 3.93E-34 | **1.11E-31** |
| **AQP8** | 16p12.1 | 0.47 | 8.18E-34 | **2.29E-31** |
| **MIR22HG** | 17p13.3 | 0.467 | 2.28E-33 | **6.30E-31** |
| **BTNL8** | 5q35.3 | 0.467 | 2.42E-33 | **6.59E-31** |
| **SI** | 3q26.1 | 0.574 | 2.60E-33 | **6.98E-31** |
| **SLC41A2** | 12q23.3 | 0.465 | 4.61E-33 | **1.22E-30** |
| **B3GNT7** | 2q37.1\|2q37.1 | 0.463 | 8.64E-33 | **2.26E-30** |
| **SLC26A3** | 7q22.3-q31.1 | 0.462 | 1.09E-32 | **2.82E-30** |
| **SPINK4** | 9p13.3 | 0.462 | 1.29E-32 | **3.29E-30** |
| **SLC30A10** | 1q41 | 0.462 | 1.32E-32 | **3.32E-30** |
| **MUC2** | 11p15.5 | 0.462 | 1.43E-32 | **3.54E-30** |
| **MOGAT2** | 11q13.5 | 0.461 | 1.60E-32 | **3.92E-30** |
| **SLC28A2** | 15q21.1 | 0.46 | 2.21E-32 | **5.35E-30** |
| **BCAS1** | 20q13.2 | 0.46 | 2.92E-32 | **6.98E-30** |
| **CA1** | 8q21.2 | 0.565 | 3.53E-32 | **8.36E-30** |
| **CLCA1** | 1p22.3 | 0.456 | 8.79E-32 | **2.05E-29** |
| **BMP2** | 20p12.3 | 0.455 | 1.40E-31 | **3.23E-29** |
| **PIGR** | 1q32.1 | 0.453 | 2.30E-31 | **5.25E-29** |
| **CCDC68** | 18q21.2 | 0.453 | 3.17E-31 | **7.15E-29** |
| **ALPI** | 2q37.1 | 0.451 | 4.63E-31 | **1.03E-28** |
| **TPSG1** | 16p13.3 | 0.448 | 1.38E-30 | **3.04E-28** |
| **PI3** | 20q13.12 | 0.442 | 9.79E-30 | **2.14E-27** |
| **EMP1** | 12p13.1 | 0.442 | 1.06E-29 | **2.28E-27** |
| **UGT2B15** | 4q13.2 | 0.442 | 1.11E-29 | **2.38E-27** |
| **GBA3** | 4p15.2 | 0.439 | 2.70E-29 | **5.70E-27** |
| **IL18** | 11q23.1 | 0.437 | 6.16E-29 | **1.29E-26** |
| **TMEM37** | 2q14.2 | 0.433 | 1.99E-28 | **4.11E-26** |
| **CLDN8** | 21q22.11 | 0.433 | 2.07E-28 | **4.25E-26** |
| **SIAE** | 11q24.2 | 0.43 | 4.28E-28 | **8.68E-26** |
| **DGKA** | 12q13.2 | 0.429 | 5.98E-28 | **1.20E-25** |
| **MUC4** | 3q29 | 0.429 | 6.95E-28 | **1.38E-25** |
| **PLA2G2A** | 1p36.13 | 0.429 | 7.45E-28 | **1.46E-25** |
| **DNASE1L3** | 3p14.3 | 0.428 | 1.04E-27 | **2.03E-25** |
| **ETHE1** | 19q13.31 | 0.427 | 1.23E-27 | **2.38E-25** |
| **CYP2C18** | 10q23.33 | 0.427 | 1.34E-27 | **2.57E-25** |
| **TMEM253** | 14q11.2 | 0.427 | 1.41E-27 | **2.66E-25** |
| **BTNL3** | 5q35.3 | 0.426 | 1.84E-27 | **3.44E-25** |
| **ITM2C** | 2q37.1 | 0.426 | 1.97E-27 | **3.65E-25** |
| **NXPE4** | 11q23.2 | 0.424 | 3.23E-27 | **5.93E-25** |
| **C4BPA** | 1q32.2 | 0.424 | 3.25E-27 | **5.93E-25** |
| **HPGD** | 4q34.1 | 0.423 | 4.24E-27 | **7.66E-25** |
| **C4BPB** | 1q32.1 | 0.422 | 5.21E-27 | **9.32E-25** |
| **FFAR4** | 10q23.33 | 0.422 | 6.49E-27 | **1.15E-24** |
| **UGT1A10** | 2q37.1 | 0.42 | 9.16E-27 | **1.60E-24** |
| **C4ORF19** | 4p14 | 0.42 | 9.20E-27 | **1.60E-24** |
| **ENKUR** | 10p12.1 | 0.42 | 1.03E-26 | **1.78E-24** |
| **BCL10** | 1p22.3 | 0.419 | 1.26E-26 | **2.15E-24** |
| **SPINK5** | 5q32 | 0.419 | 1.33E-26 | **2.25E-24** |
| **GSKIP** | 14q32.2 | 0.419 | 1.64E-26 | **2.74E-24** |
| **IFNGR1** | 6q23.3 | 0.419 | 1.64E-26 | **2.74E-24** |
| **GPA33** | 1q24.1 | 0.418 | 2.01E-26 | **3.33E-24** |
| **COL17A1** | 10q25.1 | 0.418 | 2.10E-26 | **3.44E-24** |
| **ADH1C** | 4q23 | 0.417 | 2.43E-26 | **3.92E-24** |
| **ABHD3** | 18q11.2 | 0.417 | 2.45E-26 | **3.92E-24** |
| **SLC22A18AS** | 11p15.4 | 0.417 | 2.45E-26 | **3.92E-24** |
| **F2RL1** | 5q13.3 | 0.414 | 5.60E-26 | **8.90E-24** |
| **OTOP2** | 17q25.1 | 0.514 | 5.76E-26 | **9.09E-24** |
| **PCAT18** | 18q11.2 | 0.414 | 5.97E-26 | **9.34E-24** |
| **SCNN1B** | 16p12.2 | 0.413 | 9.41E-26 | **1.46E-23** |
| **TMIGD1** | 17q11.2 | 0.511 | 1.25E-25 | **1.93E-23** |
| **ST3GAL4** | 11q24.2 | 0.412 | 1.30E-25 | **1.99E-23** |
| **LINC00520** | 14q22.3 | 0.411 | 1.57E-25 | **2.39E-23** |
| **SPDEF** | 6p21.31 | 0.408 | 3.28E-25 | **4.93E-23** |
| **TEX11** | Xq13.1 | 0.408 | 3.58E-25 | **5.35E-23** |
| **SQOR** | 15q21.1 | 0.408 | 3.83E-25 | **5.68E-23** |
| **ST6GALNAC6** | 9q34.11 | 0.407 | 4.42E-25 | **6.51E-23** |
| **C1QTNF12** | 1p36.33 | 0.407 | 4.71E-25 | **6.88E-23** |
| **ALDH1L1** | 3q21.3 | 0.407 | 4.95E-25 | **7.18E-23** |
| **CPM** | 12q15 | 0.407 | 5.10E-25 | **7.35E-23** |
| **LDHD** | 16q23.1 | 0.407 | 5.74E-25 | **8.20E-23** |
| **CAP1** | 1p34.2 | 0.406 | 7.34E-25 | **1.04E-22** |
| **CLDN23** | 8p23.1 | 0.405 | 9.42E-25 | **1.33E-22** |
| **MOB3B** | 9p21.2 | 0.404 | 1.12E-24 | **1.57E-22** |
| **NXPE1** | 11q23.2 | 0.404 | 1.14E-24 | **1.58E-22** |
| **IL1R2** | 2q11.2 | 0.404 | 1.28E-24 | **1.77E-22** |
| **REP15** | 12p11.22 | 0.402 | 1.97E-24 | **2.71E-22** |
| **PLCE1** | 10q23.33 | 0.402 | 2.02E-24 | **2.75E-22** |
| **SLC6A14** | Xq23 | 0.401 | 2.81E-24 | **3.80E-22** |
| **PADI2** | 1p36.13 | 0.4 | 3.29E-24 | **4.41E-22** |
| **INSC** | 11p15.2 | 0.4 | 3.57E-24 | **4.76E-22** |
| **F3** | 1p21.3 | 0.4 | 4.13E-24 | **5.48E-22** |
| **HEPACAM2** | 7q21.2 | 0.398 | 6.63E-24 | **8.72E-22** |
| **RHOC** | 1p13.2 | 0.397 | 7.62E-24 | **9.96E-22** |
| **SULT1B1** | 4q13.3 | 0.397 | 9.09E-24 | **1.18E-21** |
| **TNFRSF17** | 16p13.13 | 0.397 | 9.67E-24 | **1.25E-21** |
| **VNN1** | 6q23.2 | 0.396 | 1.06E-23 | **1.36E-21** |
| **PAPSS2** | 10q23.2-q23.31 | 0.395 | 1.65E-23 | **2.11E-21** |
| **P3H2** | 3q28 | 0.395 | 1.67E-23 | **2.11E-21** |
| **TPK1** | 7q35 | 0.392 | 3.42E-23 | **4.30E-21** |
| **FAM83A** | 8q24.13 | 0.392 | 3.66E-23 | **4.57E-21** |
| **SMPDL3A** | 6q22.31 | 0.39 | 6.14E-23 | **7.62E-21** |
| **CDKN1A** | 6p21.2 | 0.389 | 7.39E-23 | **9.12E-21** |
| **SEMA6D** | 15q21.1 | 0.389 | 7.77E-23 | **9.53E-21** |
| **MADCAM1** | 19p13.3 | 0.389 | 8.24E-23 | **1.00E-20** |
| **CASP10** | 2q33.1 | 0.389 | 9.06E-23 | **1.09E-20** |
| **CEACAM1** | 19q13.2 | 0.389 | 9.06E-23 | **1.09E-20** |
| **SLC44A4** | 6p21.33 | 0.388 | 1.17E-22 | **1.40E-20** |
| **ADAM6** | 14q32.33 | 0.387 | 1.20E-22 | **1.43E-20** |
| **CD55** | 1q32.2 | 0.387 | 1.31E-22 | **1.54E-20** |
| **CWH43** | 4p11 | 0.387 | 1.42E-22 | **1.68E-20** |
| **STYK1** | 12p13.2 | 0.386 | 1.82E-22 | **2.12E-20** |
| **DUOXA1** | 15q21.1 | 0.386 | 1.89E-22 | **2.19E-20** |
| **FAM118B** | 11q24.2 | 0.386 | 2.03E-22 | **2.34E-20** |
| **CLINT1** | 5q33.3 | 0.384 | 2.89E-22 | **3.32E-20** |
| **USP2** | 11q23.3 | 0.383 | 3.72E-22 | **4.25E-20** |
| **LRRC19** | 9p21.2 | 0.383 | 3.96E-22 | **4.49E-20** |
| **LGR4** | 11p14.1 | 0.38 | 9.72E-22 | **1.10E-19** |
| **FAM214B** | 9p13.3 | 0.379 | 1.09E-21 | **1.22E-19** |
| **GCG** | 2q24.2 | 0.472 | 1.11E-21 | **1.24E-19** |
| **FRMD3** | 9q21.32 | 0.379 | 1.20E-21 | **1.33E-19** |
| **ASPG** | 14q32.33 | 0.377 | 1.77E-21 | **1.96E-19** |
| **SYTL5** | Xp11.4 | 0.377 | 1.89E-21 | **2.07E-19** |
| **BCL2L15** | 1p13.2 | 0.376 | 2.77E-21 | **3.03E-19** |
| **SPPL2A** | 15q21.2 | 0.375 | 3.52E-21 | **3.82E-19** |
| **CHST5** | 16q23.1 | 0.373 | 4.92E-21 | **5.31E-19** |
| **PRR5L** | 11p13-p12 | 0.373 | 4.95E-21 | **5.32E-19** |
| **ACER3** | 11q13.5 | 0.373 | 5.43E-21 | **5.80E-19** |
| **CPNE5** | 6p21.2 | 0.372 | 6.81E-21 | **7.24E-19** |
| **BAK1** | 6p21.31 | 0.371 | 8.88E-21 | **9.39E-19** |
| **ADM** | 11p15.4 | 0.371 | 9.08E-21 | **9.55E-19** |
| **MT1M** | 16q13 | 0.371 | 9.44E-21 | **9.87E-19** |
| **RHBDL2** | 1p34.3 | 0.37 | 1.19E-20 | **1.23E-18** |
| **ENTPD8** | 9q34.3 | 0.37 | 1.25E-20 | **1.29E-18** |
| **CNNM4** | 2q11.2 | 0.369 | 1.44E-20 | **1.48E-18** |
| **RIOK3** | 18q11.2 | 0.367 | 2.32E-20 | **2.37E-18** |
| **SERPINB7** | 18q21.33 | 0.367 | 2.33E-20 | **2.38E-18** |
| **TNIP3** | 4q27 | 0.367 | 2.76E-20 | **2.80E-18** |
| **DAPP1** | 4q23 | 0.367 | 2.89E-20 | **2.91E-18** |
| **FABP2** | 4q26 | 0.366 | 3.23E-20 | **3.24E-18** |
| **EDN3** | 20q13.32 | 0.366 | 3.32E-20 | **3.32E-18** |
| **BARX2** | 11q24.3 | 0.366 | 3.39E-20 | **3.37E-18** |

**Supplementary Table 3. The top 200 genes negatively correlated with DHRS9.**

| **Correlated Gene** | **Cytoband** | **Spearman's Correlation** | **p-Value** | **q-Value** |
| --- | --- | --- | --- | --- |
| **ACVR2B** | 3p22.2 | -0.365 | 4.54E-20 | **4.46E-18** |
| **ZBTB12** | 6p21.33 | -0.343 | 9.74E-18 | **7.33E-16** |
| **EHMT2** | 6p21.33 | -0.338 | 2.94E-17 | **2.10E-15** |
| **GDPD5** | 11q13.4-q13.5 | -0.333 | 8.20E-17 | **5.60E-15** |
| **BCL11A** | 2p16.1 | -0.321 | 1.06E-15 | **6.33E-14** |
| **ZNF250** | 8q24.3 | -0.321 | 1.20E-15 | **7.08E-14** |
| **PATZ1** | 22q12.2 | -0.32 | 1.61E-15 | **9.40E-14** |
| **CEP68** | 2p14 | -0.316 | 3.28E-15 | **1.81E-13** |
| **MORC2** | 22q12.2 | -0.311 | 8.76E-15 | **4.54E-13** |
| **ANKK1** | 11q23.2 | -0.311 | 9.23E-15 | **4.75E-13** |
| **RP9P** | 7p14.3 | -0.307 | 1.99E-14 | **9.80E-13** |
| **POLR1E** | 9p13.2 | -0.307 | 2.39E-14 | **1.16E-12** |
| **PLEKHB1** | 11q13.4 | -0.305 | 3.02E-14 | **1.44E-12** |
| **ZNF696** | 8q24.3 | -0.304 | 4.29E-14 | **1.97E-12** |
| **NMNAT3** | 3q23 | -0.303 | 4.96E-14 | **2.25E-12** |
| **FXR1** | 3q26.33 | -0.302 | 6.17E-14 | **2.76E-12** |
| **SNHG32** | 6p21.33 | -0.3 | 8.27E-14 | **3.65E-12** |
| **SETD6** | 16q21 | -0.298 | 1.25E-13 | **5.41E-12** |
| **COQ8A** | 1q42.13 | -0.294 | 2.77E-13 | **1.15E-11** |
| **ZNF74** | 22q11.21 | -0.294 | 2.80E-13 | **1.15E-11** |
| **ZNRF3** | 22q12.1 | -0.293 | 3.68E-13 | **1.50E-11** |
| **LARGE2** | 11p11.2 | -0.292 | 4.19E-13 | **1.69E-11** |
| **YPEL1** | 22q11.21-q11.22 | -0.291 | 4.94E-13 | **1.98E-11** |
| **NCK2** | 2q12.2 | -0.29 | 6.09E-13 | **2.43E-11** |
| **CROCC** | 1p36.13 | -0.289 | 7.96E-13 | **3.12E-11** |
| **PACSIN3** | 11p11.2 | -0.288 | 8.52E-13 | **3.31E-11** |
| **DTNB** | 2p23.3 | -0.288 | 9.55E-13 | **3.67E-11** |
| **PHF10** | 6q27 | -0.287 | 1.07E-12 | **4.08E-11** |
| **TSPYL2** | Xp11.22 | -0.287 | 1.11E-12 | **4.20E-11** |
| **RP9** | 7p14.3 | -0.286 | 1.43E-12 | **5.28E-11** |
| **XPC** | 3p25.1 | -0.285 | 1.63E-12 | **5.98E-11** |
| **CEP250** | 20q11.22 | -0.285 | 1.66E-12 | **6.09E-11** |
| **SPINDOC** | 11q13.1 | -0.283 | 2.44E-12 | **8.70E-11** |
| **TFAP4** | 16p13.3 | -0.282 | 2.93E-12 | **1.03E-10** |
| **C11ORF95** | 11q13.1 | -0.282 | 2.95E-12 | **1.03E-10** |
| **ZXDC** | 3q21.3 | -0.282 | 2.99E-12 | **1.05E-10** |
| **ZMYM3** | Xq13.1 | -0.281 | 3.18E-12 | **1.11E-10** |
| **SORBS1** | 10q24.1 | -0.281 | 3.25E-12 | **1.13E-10** |
| **SRCIN1** | 17q12 | -0.28 | 3.64E-12 | **1.25E-10** |
| **PROX1** | 1q32.3 | -0.28 | 3.76E-12 | **1.28E-10** |
| **TUT1** | 11q12.3 | -0.28 | 3.78E-12 | **1.29E-10** |
| **SAFB** | 19p13.3 | -0.28 | 3.98E-12 | **1.34E-10** |
| **PAAF1** | 11q13.4 | -0.279 | 4.49E-12 | **1.50E-10** |
| **KANK1** | 9p24.3 | -0.279 | 4.67E-12 | **1.55E-10** |
| **PTK7** | 6p21.1 | -0.279 | 4.89E-12 | **1.62E-10** |
| **ARHGEF19** | 1p36.13 | -0.278 | 5.45E-12 | **1.79E-10** |
| **CAD** | 2p23.3 | -0.277 | 6.47E-12 | **2.12E-10** |
| **SMTN** | 22q12.2 | -0.277 | 6.51E-12 | **2.13E-10** |
| **SLC6A6** | 3p25.1 | -0.277 | 6.75E-12 | **2.19E-10** |
| **CHD6** | 20q12 | -0.277 | 6.78E-12 | **2.20E-10** |
| **TTC28-AS1** | 22q12.1 | -0.277 | 6.87E-12 | **2.22E-10** |
| **PRPF40B** | 12q13.12 | -0.277 | 7.05E-12 | **2.27E-10** |
| **TUT4** | 1p32.3 | -0.277 | 7.30E-12 | **2.35E-10** |
| **MEX3A** | 1q22 | -0.277 | 7.38E-12 | **2.37E-10** |
| **ZSCAN2** | 15q25.2 | -0.277 | 7.40E-12 | **2.37E-10** |
| **CEP131** | 17q25.3 | -0.277 | 7.41E-12 | **2.37E-10** |
| **MAP3K20** | 2q31.1 | -0.276 | 7.98E-12 | **2.55E-10** |
| **PHKA2** | Xp22.13 | -0.276 | 8.56E-12 | **2.72E-10** |
| **RNF38** | 9p13.2 | -0.276 | 8.92E-12 | **2.81E-10** |
| **WDR19** | 4p14 | -0.274 | 1.10E-11 | **3.41E-10** |
| **DNMT3A** | 2p23.3 | -0.273 | 1.39E-11 | **4.25E-10** |
| **CACNA1D** | 3p21.1 | -0.273 | 1.39E-11 | **4.26E-10** |
| **SETDB1** | 1q21.3 | -0.273 | 1.40E-11 | **4.26E-10** |
| **TRMT10B** | 9p13.2 | -0.272 | 1.73E-11 | **5.20E-10** |
| **ADNP** | 20q13.13 | -0.271 | 1.92E-11 | **5.75E-10** |
| **SLC2A12** | 6q23.2 | -0.271 | 1.97E-11 | **5.86E-10** |
| **ZNF766** | 19q13.41 | -0.271 | 1.99E-11 | **5.91E-10** |
| **CELSR2** | 1p13.3 | -0.271 | 2.17E-11 | **6.41E-10** |
| **CUL7** | 6p21.1 | -0.27 | 2.21E-11 | **6.54E-10** |
| **SLC41A3** | 3q21.2-q21.3 | -0.27 | 2.47E-11 | **7.28E-10** |
| **CDON** | 11q24.2 | -0.269 | 2.84E-11 | **8.30E-10** |
| **CSNK1E** | 22q13.1 | -0.269 | 2.90E-11 | **8.43E-10** |
| **TOP1MT** | 8q24.3 | -0.268 | 3.23E-11 | **9.33E-10** |
| **ZNF84** | 12q24.33 | -0.268 | 3.24E-11 | **9.36E-10** |
| **TGIF2** | 20q11.23 | -0.267 | 4.35E-11 | **1.24E-09** |
| **RCN1** | 11p13 | -0.266 | 4.59E-11 | **1.31E-09** |
| **UBTF** | 17q21.31 | -0.266 | 5.06E-11 | **1.43E-09** |
| **FOXP4** | 6p21.1 | -0.265 | 5.48E-11 | **1.54E-09** |
| **PIP4K2B** | 17q12 | -0.265 | 5.68E-11 | **1.59E-09** |
| **TTLL1** | 22q13.2 | -0.265 | 6.12E-11 | **1.70E-09** |
| **IGF1R** | 15q26.3 | -0.264 | 6.69E-11 | **1.84E-09** |
| **ZNF251** | 8q24.3 | -0.264 | 6.82E-11 | **1.87E-09** |
| **ATIC** | 2q35 | -0.264 | 7.19E-11 | **1.97E-09** |
| **HNRNPH3** | 10q21.3 | -0.264 | 7.28E-11 | **1.99E-09** |
| **ZNF517** | 8q24.3 | -0.263 | 7.44E-11 | **2.03E-09** |
| **NOTCH1** | 9q34.3 | -0.263 | 7.81E-11 | **2.12E-09** |
| **CADM4** | 19q13.31 | -0.263 | 8.28E-11 | **2.23E-09** |
| **ZNF445** | 3p21.31 | -0.262 | 8.97E-11 | **2.41E-09** |
| **RUSC2** | 9p13.3 | -0.262 | 9.34E-11 | **2.50E-09** |
| **HNRNPU** | 1q44 | -0.261 | 1.08E-10 | **2.85E-09** |
| **THRSP** | 11q14.1 | -0.261 | 1.14E-10 | **2.98E-09** |
| **PLCG1** | 20q12 | -0.261 | 1.17E-10 | **3.05E-09** |
| **C11ORF74** | 11p12 | -0.261 | 1.21E-10 | **3.14E-09** |
| **PMS2CL** | 7p22.1 | -0.261 | 1.22E-10 | **3.17E-09** |
| **ING5** | 2q37.3 | -0.26 | 1.26E-10 | **3.26E-09** |
| **TCEAL4** | Xq22.2 | -0.26 | 1.33E-10 | **3.42E-09** |
| **SMARCC2** | 12q13.2 | -0.26 | 1.44E-10 | **3.69E-09** |
| **ASXL1** | 20q11.21 | -0.259 | 1.48E-10 | **3.78E-09** |
| **PLEKHA8P1** | 12q12 | -0.259 | 1.51E-10 | **3.85E-09** |
| **KBTBD6** | 13q14.11 | -0.259 | 1.56E-10 | **3.97E-09** |
| **NPTXR** | 22q13.1 | -0.258 | 1.75E-10 | **4.42E-09** |
| **CNTRL** | 9q33.2 | -0.258 | 1.79E-10 | **4.51E-09** |
| **RNF20** | 9q31.1 | -0.258 | 1.99E-10 | **4.95E-09** |
| **PRPF6** | 20q13.33 | -0.257 | 2.08E-10 | **5.14E-09** |
| **CNPY3** | 6p21.1 | -0.257 | 2.10E-10 | **5.18E-09** |
| **CSNK2A2** | 16q21 | -0.257 | 2.15E-10 | **5.29E-09** |
| **CDCA7L** | 7p15.3 | -0.257 | 2.19E-10 | **5.39E-09** |
| **TSNARE1** | 8q24.3 | -0.257 | 2.23E-10 | **5.46E-09** |
| **SMYD5** | 2p13.2 | -0.257 | 2.24E-10 | **5.48E-09** |
| **PHC1** | 12p13.31 | -0.257 | 2.29E-10 | **5.59E-09** |
| **IRF2BP2** | 1q42.3 | -0.257 | 2.31E-10 | **5.65E-09** |
| **ANKRD27** | 19q13.11 | -0.256 | 2.64E-10 | **6.41E-09** |
| **MED12** | Xq13.1 | -0.256 | 2.82E-10 | **6.78E-09** |
| **KIF3C** | 2p23.3 | -0.255 | 2.97E-10 | **7.10E-09** |
| **DDX42** | 17q23.3 | -0.255 | 2.97E-10 | **7.10E-09** |
| **RNF216** | 7p22.1 | -0.255 | 3.15E-10 | **7.45E-09** |
| **ETNK2** | 1q32.1 | -0.255 | 3.21E-10 | **7.57E-09** |
| **PMFBP1** | 16q22.2 | -0.254 | 3.41E-10 | **8.02E-09** |
| **LDLRAD3** | 11p13 | -0.254 | 3.43E-10 | **8.07E-09** |
| **CDK5RAP2** | 9q33.2 | -0.254 | 3.62E-10 | **8.49E-09** |
| **USP11** | Xp11.3 | -0.254 | 3.63E-10 | **8.50E-09** |
| **SUGP1** | 19p13.11 | -0.254 | 3.87E-10 | **8.98E-09** |
| **PYCR2** | 1q42.12 | -0.253 | 3.93E-10 | **9.12E-09** |
| **ASIC1** | 12q13.12 | -0.253 | 3.94E-10 | **9.12E-09** |
| **RPIA** | 2p11.2 | -0.253 | 4.12E-10 | **9.51E-09** |
| **NCL** | 2q37.1 | -0.253 | 4.33E-10 | **9.95E-09** |
| **LRIG1** | 3p14.1 | -0.253 | 4.54E-10 | **1.04E-08** |
| **YEATS2** | 3q27.1 | -0.253 | 4.56E-10 | **1.04E-08** |
| **GAS8** | 16q24.3 | -0.252 | 4.68E-10 | **1.07E-08** |
| **XRCC1** | 19q13.31 | -0.252 | 4.80E-10 | **1.10E-08** |
| **WDR35** | 2p24.1 | -0.252 | 4.83E-10 | **1.10E-08** |
| **LSM14B** | 20q13.33 | -0.252 | 5.42E-10 | **1.23E-08** |
| **SUPT3H** | 6p21.1 | -0.251 | 5.55E-10 | **1.26E-08** |
| **ZNF629** | 16p11.2 | -0.251 | 5.58E-10 | **1.26E-08** |
| **CGNL1** | 15q21.3 | -0.251 | 5.61E-10 | **1.26E-08** |
| **ZNF202** | 11q24.1 | -0.251 | 5.66E-10 | **1.27E-08** |
| **TWNK** | 10q24.31 | -0.251 | 5.78E-10 | **1.30E-08** |
| **RBM15B** | 3p21.2 | -0.251 | 6.01E-10 | **1.34E-08** |
| **RTL10** | 22q11.21 | -0.251 | 6.05E-10 | **1.35E-08** |
| **ZNF37BP** | 10q11.21 | -0.25 | 6.56E-10 | **1.45E-08** |
| **SNTB1** | 8q24.12 | -0.25 | 6.62E-10 | **1.46E-08** |
| **PRKCSH** | 19p13.2 | -0.25 | 6.99E-10 | **1.54E-08** |
| **ZBTB49** | 4p16.3 | -0.25 | 7.08E-10 | **1.56E-08** |
| **SLC5A6** | 2p23.3 | -0.25 | 7.45E-10 | **1.63E-08** |
| **WNK2** | 9q22.31 | -0.249 | 7.51E-10 | **1.64E-08** |
| **ANKH** | 5p15.2 | -0.249 | 7.88E-10 | **1.71E-08** |
| **GNAS** | 20q13.32 | -0.248 | 8.83E-10 | **1.90E-08** |
| **KANSL3** | 2q11.2 | -0.248 | 1.00E-09 | **2.13E-08** |
| **RNF220** | 1p34.1 | -0.247 | 1.19E-09 | **2.49E-08** |
| **ABHD1** | 2p23.3 | -0.246 | 1.29E-09 | **2.70E-08** |
| **CBFA2T2** | 20q11.21-q11.22 | -0.246 | 1.40E-09 | **2.91E-08** |
| **ZNF875** | 19q13.12 | -0.245 | 1.46E-09 | **3.02E-08** |
| **DHX57** | 2p22.1 | -0.245 | 1.57E-09 | **3.25E-08** |
| **GNAZ** | 22q11.22-q11.23 | -0.245 | 1.61E-09 | **3.32E-08** |
| **NOL8** | 9q22.31 | -0.244 | 1.70E-09 | **3.50E-08** |
| **SBK1** | 16p12.1 | -0.244 | 1.71E-09 | **3.52E-08** |
| **CBX4** | 17q25.3 | -0.244 | 1.72E-09 | **3.52E-08** |
| **LRRC2** | 3p21.31 | -0.244 | 1.81E-09 | **3.70E-08** |
| **NFYB** | 12q23.3 | -0.243 | 2.02E-09 | **4.09E-08** |
| **DRD2** | 11q23.2 | -0.243 | 2.02E-09 | **4.09E-08** |
| **INHBB** | 2q14.2 | -0.243 | 2.02E-09 | **4.09E-08** |
| **ZNF638** | 2p13.3-p13.2 | -0.243 | 2.03E-09 | **4.11E-08** |
| **SEMA4C** | 2q11.2 | -0.243 | 2.07E-09 | **4.17E-08** |
| **YTHDC1** | 4q13.2 | -0.243 | 2.09E-09 | **4.20E-08** |
| **FUS** | 16p11.2 | -0.243 | 2.14E-09 | **4.30E-08** |
| **BCORL1** | Xq26.1 | -0.243 | 2.24E-09 | **4.48E-08** |
| **HSDL1** | 16q24.1 | -0.242 | 2.33E-09 | **4.64E-08** |
| **ESYT3** | 3q22.3 | -0.242 | 2.34E-09 | **4.64E-08** |
| **FRS3** | 6p21.1 | -0.242 | 2.48E-09 | **4.91E-08** |
| **PHLDB3** | 19q13.31 | -0.242 | 2.51E-09 | **4.97E-08** |
| **FAXC** | 6q16.2 | -0.242 | 2.55E-09 | **5.03E-08** |
| **WWC3** | Xp22.2 | -0.241 | 2.71E-09 | **5.32E-08** |
| **KCNIP3** | 2q11.1 | -0.241 | 2.75E-09 | **5.40E-08** |
| **PABPC4** | 1p34.3 | -0.241 | 3.04E-09 | **5.91E-08** |
| **TP53BP1** | 15q15.3 | -0.24 | 3.10E-09 | **6.01E-08** |
| **GRIPAP1** | Xp11.23 | -0.24 | 3.10E-09 | **6.01E-08** |
| **SART1** | 11q13.1 | -0.24 | 3.12E-09 | **6.04E-08** |
| **ZBTB2** | 6q25.1 | -0.24 | 3.29E-09 | **6.34E-08** |
| **TTC25** | 17q21.2 | -0.24 | 3.45E-09 | **6.64E-08** |
| **NCOA5** | 20q13.12 | -0.239 | 3.62E-09 | **6.93E-08** |
| **IDI2** | 10p15.3 | -0.239 | 3.68E-09 | **7.04E-08** |
| **HDAC5** | 17q21.31 | -0.239 | 3.91E-09 | **7.47E-08** |
| **COX19** | 7p22.3 | -0.239 | 3.93E-09 | **7.50E-08** |
| **DLG5-AS1** | 10q22.3 | -0.239 | 3.96E-09 | **7.53E-08** |
| **WRNIP1** | 6p25.2 | -0.238 | 4.28E-09 | **8.10E-08** |
| **RPL11** | 1p36.11 | -0.238 | 4.68E-09 | **8.80E-08** |
| **RBM4B** | 11q13.2 | -0.237 | 5.26E-09 | **9.78E-08** |
| **BCAM** | 19q13.32 | -0.237 | 5.30E-09 | **9.83E-08** |
| **UBIAD1** | 1p36.22 | -0.237 | 5.45E-09 | **1.01E-07** |
| **NKD1** | 16q12.1 | -0.236 | 5.78E-09 | **1.07E-07** |
| **RPL37** | 5p13.1 | -0.236 | 5.81E-09 | **1.07E-07** |
| **NRK** | Xq22.3 | -0.236 | 5.86E-09 | **1.08E-07** |
| **LGR5** | 12q21.1 | -0.236 | 6.39E-09 | **1.18E-07** |
| **VAV2** | 9q34.2 | -0.236 | 6.44E-09 | **1.18E-07** |
| **EIF2B4** | 2p23.3 | -0.236 | 6.49E-09 | **1.19E-07** |
| **PHYHIP** | 8p21.3 | -0.236 | 6.64E-09 | **1.22E-07** |
| **LZTS2** | 10q24.31 | -0.235 | 6.84E-09 | **1.25E-07** |
| **TMEM63A** | 1q42.12 | -0.235 | 6.98E-09 | **1.27E-07** |
| **ILF3** | 19p13.2 | -0.235 | 7.04E-09 | **1.28E-07** |
| **PHF20** | 20q11.22-q11.23 | -0.235 | 7.10E-09 | **1.29E-07** |
